# Polyelectrolyte Precipitation: A New Green Chemistry Approach to Recover Value-Added Proteins from Different Sources in a Circular Economy Context

**DOI:** 10.3390/molecules27165115

**Published:** 2022-08-11

**Authors:** Ricardo Gómez-García, Ana A. Vilas-Boas, Ana Martins Vilas-Boas, Débora A. Campos, Manuela Pintado

**Affiliations:** Universidade Católica Portuguesa, CBQF-Centro de Biotecnologia e Química Fina-Aboratório Associado, Escola Superior de Biotecnologia, Rua Diogo Botelho 1327, 4169-005 Porto, Portugal

**Keywords:** green process, polyelectrolytes, protein recovery, circular economy, sustainability, ionic precipitation, food waste, plant-based proteins

## Abstract

Proteins have always been vital biological molecules used for industrial purposes, human nutrition and health. Nowadays, seeking new alternatives and sources of these biomolecules is becoming an increasing research trend derived from the present consumer awareness between food consumption and health promotion, but also on environmental sustainability. Although there are different consolidated/traditional downstream processes to obtain proteins, such as chromatography tools, alkali hydrolysis, precipitation by inorganic salts and organic solvents, their industrial-scale application still demands urgent innovation due to the poor recovery yields, high costs and time-consuming steps, environmental impact as well as some toxic concerns. Polyelectrolyte precipitation represents a green, innovative alternative for protein recovery; however, there are reduced data regarding its pilot or industrial-scale application. In this literature work, the action mechanism and principles with regards to its functionality and insights for its application on a big scale are reviewed. Overall, this review discusses the novelty and sustainability of protein precipitation by polyelectrolytes from different sources against traditional techniques as well as highlights the relationship between protein source, production relevance and bioactive properties that are key factors to maximize the application of this extractive method on a circular economy context.

## 1. Introduction

The global demand for innovative and functional foods and materials has increased over the ten years and, with a fast-growing population, the need for alternative resources emerges alongside the large generation of biomass waste. Food waste (FW) accounts for up to 50% of the total waste and in Europe is composed of 40% vegetables and fruit, 33% pasta and bread and 25% dairy products and meat residues [1]. The generation of FW is inevitable but can be avoided and reduced by creating methods which can valorize FW toward a sustainable bioeconomy, accomplishing the United Nations sustainable development goal of 2030 to reduce by food waste production by 50%. In 2019, there was a global generation of 931 million tons of food waste, according to the UN Environment Program Food Waste Index report [2].

Biorefinery is a concept in which FW is exploited to its integral potential and converted into value-added bioactive molecules and ingredients such as carbohydrates, proteins and bio-based enzymes, biofuels, biopolymers, bio-based molecules and materials [3]. This concept enters in the circular bioeconomy approach which encompasses the production of value-added products in a sustainable and integrated manner, while generating zero waste and reducing/eliminating waste management problems and related costs by employing green methodologies and technologies for FW valorization and recycling [2,4].

These value-added bioactive molecules can be primary constituents such as sugars, amino acids and proteins; and secondary constituents such as alkaloids, flavonoids, tannins, phenolic compounds, among others [5]. They can be applied in medicine, nutraceuticals, cosmetics, flavors/fragrances, food additives, antimicrobials, etc. [6]. Primary molecules such as proteins are gaining great industrial interest; however, the current challenge to obtain them is to create and establish environmentally friendly downstream approaches to separate, extract, purify and collect these molecules from FW throughout the production supply chain [6,7]. Many researchers have developed techniques such as alkali treatments, solid-state fermentation and enzymatic-assisted extraction to maximize structural degradation of FW and optimize the extraction yields of proteins by applying aqueous two-phase systems and chromatography techniques, among several traditional techniques such as salt and ethanolic precipitations [5]. However, due to the present disadvantages from these processes related to high-cost, complexity, null scalability and possible protein degradation, the green extraction, purification and modification of proteins originated from agro-industrial waste could promote the development of new sustainable approaches within the bioeconomy and circular economy scope [8]. Among green processes, the protein precipitation by polyelectrolytes (PEP) takes advantage of the capacity to form nonsoluble complexes between the polyelectrolyte and proteins by natural physico-chemical interactions at soft temperatures in heterogeneous and aqueous environments just as similar as those occurring in biological and natural systems, being an easily scaled-up, environmentally friendly and low-cost tool [8,9]. In this regard, polyelectrolyte-protein complexes (Poly-Pro) are considered to be a group of self-assembly molecules that form a complex of oppositely charged ions in aqueous solutions, including polyelectrolytes that form upon complexation between oppositely charged polyelectrolytes (polyelectrolyte-polyelectrolyte complex), and flocculation of oppositely charged particles and polyelectrolytes. Polyelectrolytes (Polys) are macromolecules containing several functional groups, such as carboxylic and sulphonic, which are electrically charged or can acquire an electric charge under particular conditions [9]. They can be synthetic, such as polyacrylic acids, which are not biodegradable but undergo degradation under certain physico-chemical conditions, or natural, such as polysaccharides, which are eco-friendly and can be discarded. Their solubility in water depends on the environmental conditions (e.g., pH), which affect the charge of the functional groups. Variations on the pH promote changes in the ionization state of the hydrophilic groups, producing intra- and interchain repulsions which sustain the Poly in extended conformation and as water-soluble [8,10]. If in the presence of a neutral Poly, its chains interact with each other and can form insoluble aggregates [11]. For example, oppositely charged Polys (polycations or polyanions) solubilized in water form complexes that are categorized mainly into three different classes: (i) soluble complexes (macroscopically homogeneous systems with no aggregation), (ii) systems made of turbid colloidal with suspended complex particles in the transition range to the split phase and (iii) two-phase systems of supernatant and precipitated complex, which is easily recovered as a solid [12].

Very limited literature has been reported on the application of PEP for plant-protein extraction. Based on the eco-innovativeness, disruptive approach, simple features and reproducibility, the PEP could lead to industries making strategic decisions toward its application and investment for the processing of FW to recover important value-added proteins. Therefore, the aims of this review are to discuss and highlight remarkable outcomes from PEP and its advantages, contributing with a better understanding of the theoretical principles of PEP and related imperative factors (such as technological, molecular and biochemical interactions) to be considered when exploring its great potential for future full consolidation as a protein-extraction method.

## 2. Polyelectrolyte Definition and Characterization

By definition, Polys are any macromolecules with a high number of functional groups that are electrically charged or acquire electric charge under certain conditions [9]. For instance, when Polys are dissolved in a suitable polar solvent (usually water), they spontaneously acquire or can acquire electric charge through their functional groups, which are covalently linked to them, forming either a positive (polycation) or negative (polyanions) polymeric chain; if both negative and positive groups occur, they are called polyampholytes [13]. The Polys charge is neutralized by oppositely charged, smaller counter ions that tend to preserve the neutrality. If any Poly’s solution contains a positive charge, small, negatively charged ions can accompany it. Similarly, negatively charged materials can be accompanied by small positively charged ions [9,14]. For Polys, the electrostatic interactions and solubility are main variables that influence their structure. The four stages influencing the Polys’ solubility that are attributed to the conformation in multivalent salt solutions are: (I) Polys that dissolve in water can adopt an extended conformation, (II) divalent counter ions will cause ion-bridging between two ionic sites on the polymer chain and the Polys remain soluble, (III) high volume of charge screening causes precipitation of Polys from solution with increased concentrations of salt and (IV) further increase in the concentration of salt will redissolve the Polys in solution as a result of high-charge screening.

The main groups present in the Poly are carboxylic and sulphonic groups. The importance of this composition is to know when the Poly has the necessary conditions to interact with the molecule of interest, such as proteins [9]. In their uncharged state, Polys behave like any other macromolecule; however, the ionic groups’ dissociation causes changes in their chemical and physical properties [14]. These properties, unlike uncharged molecules, are their great water solubility, their ability to swell and bind large amounts of water, and their ability to interact strongly with oppositely charged macromolecules [13]. There are different types of Polys (strong: pH-independent charge or weak: pH-dependent charge). For example, for weak ones, these changes are related to the electrostatic interactions and, hence, are mainly sensitive to the pH and the amount and type of electrolytes present in the solution [10,13]. The solubility of Polys in water depends on the state of charge of ionic groups and, therefore, on the environmental conditions affecting this state, expressed as the pH. The pH variations in the medium produce changes in the Polys’ ionic groups. When the Polys are electrically charged, intra- and interchain repulsions are produced, which maintain the macromolecule in an extended conformation and, therefore, are water-soluble. When the Polys are neutral, their structure can interact with each other and eventually form insoluble aggregates. Other properties, such as viscosity ionization constant, ionic strength and diffusion coefficient, can also affect the Poly characteristics [13,14].

**Figure 1:**
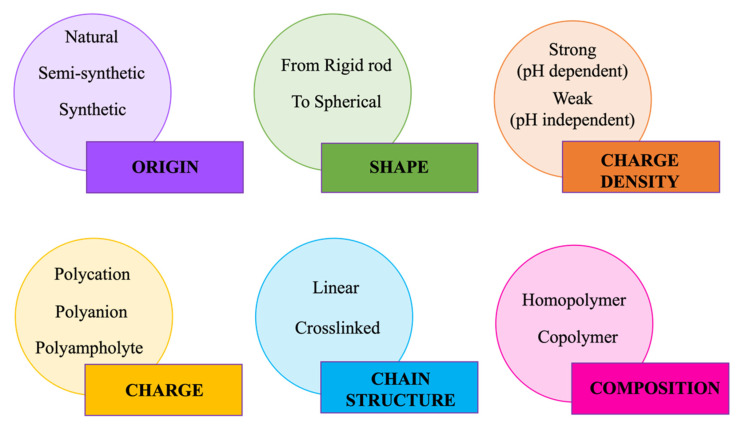
Classification of polyelectrolytes (Polys).

The Polys are classified according to some of their characteristics (Figure 1) mainly based on the origin, the charge and the composition. The most important characteristic in the development of methods for protein recovery are the use of natural and nontoxic Polys. The synthetic Polys are obtained by chemical synthesis and cannot be discarded in the environment because they are not biodegradable, and some of them are toxic and carcinogenic [9]. On the other hand, the natural Polys are environmentally friendly. Both synthetic and natural Polys are already produced and sold on a large scale. Examples of common synthetic polyelectrolytes are poly (diallyldimethyl ammonium chloride), polystyrene sulfonate, polyacrylic acid polyallylamine and their salts. Common natural Polys are pectin, alginate, carboxymethyl cellulose, carrageenan, hyaluronic acid, chitosan, dextran, gellan gum, xanthan gum and polypeptides. One of the main advantages of using natural Polys is that they can be obtained from sustainable sources such as the by-products from the agri-food industry.

Along with proteins, the polysaccharides are the major macromolecules in biological systems, and they are electrically charged with strong- or weak-acid or -basic groups. All polysaccharides are polydisperse, containing molecules with different degrees of polymerization; hence, they can be organized by structure, by shape, by monomeric units and by charge (neutral, anionic and cationic) [10]. The charge has a significant effect on the ionic polysaccharides since these macromolecules adopt an extended shape (due to Coulombic repulsion) and thus impart high viscosity to their solutions [10]. The commonly used natural polyanions are polysaccharides with polymers of sugar acid (e.g., alginate and pectin) [15], carboxyl groups that are substituted through a chemical reaction (e.g., carboxy-methylcellulose) [16] and sugar units biosynthesized with anionic substituents such as sulfate groups (e.g., carrageenan) [17]. Moreover, at pH > 4.5, due to the deprotonation of O-acetyl and pyruvyl residues, the xanthan gum turns into a polyanion and is used in these conditions [18]. This negative charge increases the water solubility and polarity; however, the intrinsic charge weakens intermolecular associations between polymer chains due to repulsion [10].

Pectin is an anionic, soluble heterogeneous polysaccharide containing linear chains of α-(1 → 4)-D-galacturonic acid residues and 1,2-D-rhamnose with D-galactose and D-arabinose side chains [19]. Apple pomace and orange peels and pomace are the two major sources of pectin, although pectin is present in the cell wall of most plants [20]. Therefore, it is possible to use pectin polysaccharides extracted from a food industry by-product in a manner similar to Polys. Regarding alginate and carrageenan, they are a natural ingredient that comes mainly from seaweed. Alginate is a linear, unbranched polymer, containing α-L-guluronic acid (G) and β-D-mannuronic acid (M) bonded by 1 → 4 linkages; therefore, it is a highly anionic and very polar polymer [21]. The physical properties of alginate depend on a number of different key factors in which M and G contents play a crucial part. Around 200 different categories of alginates have been identified and extracted from nature and the ratio of mannuronate to guluronate (M/G) varies significantly depending on the source organism and tissue from which it is being isolated and also the season when it was harvested. It can be extracted up to 40% dry matter (DM) of alginate from the cell wall of brown seaweed. The seaweed is a renewable biomass due to its abundant availability around the world, and the production process is considered biodegradable, biocompatible and environment-friendly [22]. Industrial-scale alginate production commenced in 1929, and it is estimated that there is a total of at least 30,000 tons/year of commercial alginate production. The production is predominantly derived from brown seaweed such as genera Laminaria, Macrocystis and Ascophyllum. In addition to seaweed, alginate can also be extracted from bacteria species such as Azotobacter spp. and Pseudomonas spp. [23]. Carrageenan is a collective term for sulfated polysaccharides that could be obtained from diverse species of red seaweed of the family Rhodophyceae [10]. They are composed by alternated galactopyranosyl dimer units linked by alternated β-(1-4) and α-(1-3) glycosidic bonds, and the sugar units are sulfated [24]. It can be classified as lambda (λ), kappa (κ) or iota (ι) according to their capacity to form gel [25]. Therefore, different seaweeds produce different types of carrageenans; however, one of the types is always the more predominant. The major commercial sources are Gigartina spp., Eucheuma cottonii, Chondrus crispus, Hypnea spp. and Eucheuma spinosum.

Regarding polycationic polysaccharides, there are only a few natural compounds available in the market. Chitosan is a well-established cationic polymer in nature because their amino group confers a positive charge when it is dissolved in an acidic solution. Chitosan, the N-deacetylation product of chitin, is one of the most widely occurring polymers in nature: it can be found in a range of eukaryotic species such as crustacea, insects and fungi. Chitosan is a linear polysaccharide, composed of glucosamine and N-acetyl glucosamine units via β-(1 → 4) linkages, randomly or block distributed throughout the biopolymer chain, depending on the preparation method to derive chitosan from chitin [26]. In acidic conditions, chitosan suits the only polysaccharide with high positively charged density, due to the protonation of amino groups. Moreover, chitosan has been proven to have other special properties such as nontoxicity and biodegradability [27]. Shrimp and crabs are the most common sources cited in the literature as the raw material for chitosan preparation, while other species such as lobster, crayfish and oyster have also been utilized. Seafood industries produce a large amount of waste (shells) with a high environmental impact. These worthy wastes can be converted into an added-value product—chitosan. Different crustacean by-products show different contents of chitin; however, crustacean shell waste on average consists of 20–30% (*w*/*v*) of chitin [28]. On a commercial scale, chitin/chitosan is mostly extracted chemically from shrimp shells.

Despite starch not being described as a polyelectrolyte, it could be another important polysaccharide that assumes a positive surface charge when dispersed in water, and can form complex networks between oppositely charged chains, lipids and proteins [29]. Amylose and amylopectin are two polymers that constitute starch. Amylopectin possesses a branched structure with α-1–4 as well as α-1–6 glycosidic linkages, while amylose possesses a linear structure with α-1–4 glycosidic linkage [30]. The major commercial sources of starch are corn (64%), cassava, sweet potato and potato. Although starch obtained from conventional sources has frequent usage, its extraction from unconventional sources offers an attractive alternative. Unconventional sources of starch include unripe fruits and their by-products as peels and seeds, rhizomes (ginger, turmeric, lotus), cereals (amaranthas, millet), pseudocereals (buckwheat), legumes (lima bean, navy bean, pea, lentil), nuts (horse chestnut, water chestnut) [31].

## 3. Polyelectrolyte Complex

**Figure 2:**
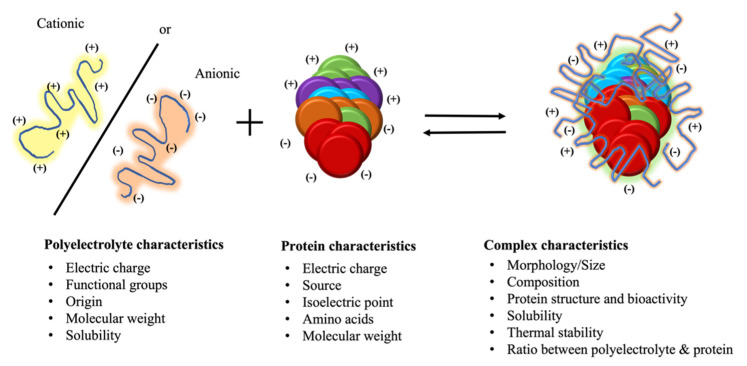
Schematic representation of the key characteristics of polyelectrolytes and proteins to develop the complex formation.

Research studies have described the interactions and phase behavior of complexes encompassing proteins and Polys, discussing the influence of ionic strength, Polys’ characteristics (size, length and charge groups) and proteins’ charge density on the complex formation. Also, phase behavior of Poly–Pro complexes is strongly dependent on the electrostatic interactions, which in turn are directed by several factors, such as Polys charge density, surface charge distribution of the protein and the solvent conditions (pH, ionic strength). Additionally, the bulk concentrations of both the protein and the Polys affect the composition and the net charge of the microscopic complexes, thereby affecting the intercomplex interactions. As general rules, good Polys for protein precipitation by complex formation should have free electrically charged groups (ligand coupling), not interact with the impurities in the system (avoid nonspecific coprecipitation), preserve the structures (secondary and tertiary) of the protein without affecting the biological activity, give high yields and good purification factors and be commercially available, cheap and natural. Polys strongly interact with proteins of the opposite electrical charge to form complexes according to the conditions of the medium. Therefore, the result of the electrostatic interaction (Coulomb’s interactions) is the formation of a Poly–Pro complex (Figure 2). These complexes cause the formation of two phases: the dilute phase and the concentrated complex phase or in a compact gel or precipitate solid [9,32]. The mutual electrostatic interactions create the Poly–Pro complex, which are predominate between polycations and polyanions upon mixing aqueous solutions of oppositely charged Polys, leading to the formation of complexes, which aggregate up to reach sizes and superficial properties that lead to phase separation [10]. The formation, properties and applications of Poly–Pro complexes have been widely described in the last decades [8,9,17,32]. The use of natural polysaccharides that have electrically charged groups increased the number of available Polys and, therefore, the possibilities to form Poly-Pro complexes [9]. The complex formation between polysaccharides and proteins is a phenomenon that allows for the reaching of the energy equilibrium, leading to a decrease in the total electrostatic free energy of the complex [10]. The electrostatic interaction of proteins with natural PE may result in amorphous precipitates, complex coacervate, gels, fibers or the formation of soluble complexes. Generally, the complex formation is driven mainly by the decrease in free energy resulting from a positive enthalpic contribution occurring from the electrostatic interactions between the biopolymers, and the negative entropic contribution arises from the release of water molecules due to the compaction of biopolymers. Other interactions, such as electrostatic and short-range, e.g., hydrophobic, van der Waals forces or hydrogen bonds, might be secondarily involved in the formation of the protein–polysaccharide complexes [10].

**Figure 3:**
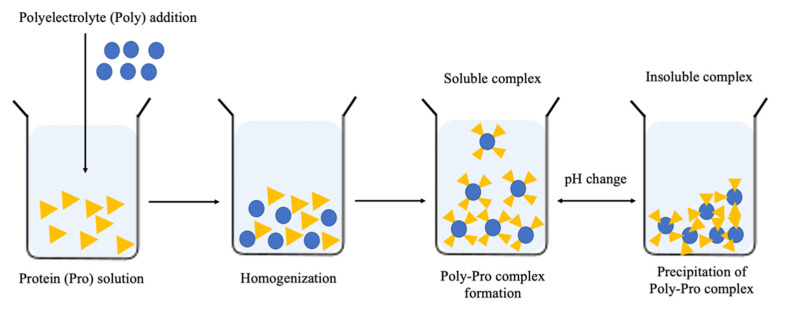
Schematic representation of polyelectrolyte-protein complex formation according to Campos et al. [10].

The Poly-Pro attractions were affected by physicochemical parameters, such as pH, the ionic strength, the number and distribution of charged sites on the protein surface, the composition and quantity of the polyelectrolyte, the charge density and the stiffness of the polysaccharide chain. Generally, the interaction happens when the pH value is far from the isoelectric point (pI) of the protein in order to avoid a neutral charge and increase the electrical (positive or negative) charge, obtaining a better interaction with the polyelectrolyte. Thus, this indicates the pH value in which the Poly–Pro complex formation is favorable to take place. Generally, the complex formation occurs within a pH range; therefore, out of that range, the soluble complexes become insoluble Poly–Pro complexes due to the charge neutralization. Therefore, the change of pH could transform the Poly–Pro complex formed by the electric charges into soluble or insoluble (Figure 3). Applying a simple centrifugation force makes it possible to separate the complex from the protein from the solution.

The main application of the Polys with proteins includes: (1) protein separation, (2) stabilization of enzymes, (3) modification of protein-substrate affinity and (4) electrostatic interactions between proteins and nucleic acids.

## 4. Perspectives on Sustainable Protein Sources

Proteins with or without biological activity (enzymes) are widely used by different industries, traditionally categorized into three sectors: technical, food and feed proteins/enzymes. Technical enzymes are applied in detergent, leather, textile and personal care industries, while food enzymes are applied for dairy, brewing, beverages and baking, and feed enzymes, usually for animal feeds [33]. In this matter, the global animal and plant-based protein market size is projected to exceed USD 70.7 billion by 2025 at a CAGR of 6.0% from 2019 [34], and the industrial enzymes are expected to increase their global demand to USD 14.7 billion by 2025, where the technical enzymes account for USD 1.5 billion by 2026, feed enzymes for USD 1.9 billion by 2025 and food enzymes for USD 3.6 billion by 2026 [35]. Consequently, the encouragement to increase innovations on proteins with functional properties as well as to expand consumer knowledge and literacy about good nutrition and health claims are indispensable. Moreover, the current trends of vegetarianism, veganism and flexitarianism have been supporting an enormous social awareness associated with preserving the environment, animal protection and a high demand of nonanimal proteins, and such trends represent rapid, interesting, rising topics for enterprises [36].

Different correlations on meat proteins’ consumption with health issues have been found due to the high use of antibiotics and hormones in livestock feed. Compared with animal sources, vegetable plant sources have lower protein content; for example, protein concentrates in a dry basis are 27% for milk, 53% for eggs, 37–83% for meats and 58–90% for sea animals, and protein concentration from vegetal raw materials is around 22–40% in legumes, 8–18% in cereals, 4–20% in nuts, 18–32% in different seeds and less than 10% in tubers. The concentration of protein in novel sources such as algae (16–66%), mushrooms (20–30%) and insects (30–60%) are also promising sources of proteins [37,38,39]. However, plant-based proteins are currently the most preferable segment due to their high nutritional quality, hypo-allergenicity, gluten-free and cholesterol-free compositions, lower saturated fatty acids content, affordability, high availability and versatility, and they also exhibit a wide array of techno- and biofunctional properties in comparison with proteins from animal and dairy sources, providing human health benefits [40,41]. In this context, the main sources of plant-based proteins for food formulations are soy proteins (63.3%), followed by wheat (46.8%), pea (40.2%), rice (7.2%) and vegetable proteins (4.7%) [42].

On the other hand, agri-food industries annually generate approximately 1.3 billion tons of FW, with an estimated economic loss of USD 990 billion. Specifically, in 2017, Europe generated 96 million tons of fruits and vegetables, corresponding to 8.5% of the total global production, and around 30% of such production was discarded as waste [43]. In Portugal, around 17%, equivalent to about 1 million tons per year, of the edible parts of the food produced for human consumption are wasted or lost, in which 42% of this waste comes from fruits and vegetables [44]. Processing FW and by-products include damaged crops, pomaces, leaves, seeds, peels, cores, brans, oilseed cakes, molasses, among others. These fruit and vegetable by-products require serious waste management, recycling and valorization processes, depending on their nature and quantity, but most of these food by-products could be valorized in order to create revenue streams [5,45]. Several agri-food by-products contain a high content of proteins, carbohydrates, lipids and other bioactive compounds including phenolics, dietary fibers, alkaloids and pigments [2,46]. The recovery of valuable compounds, such as proteins from these by-products, can improve global sustainability related with food, environment and economy, as well as meet with their increasing consumer and industrial demands. This approach is also associated with circular bioeconomy since the exhaustive energy consumption from fossil fuels and their consequent environmental pollution highly advise us to transition from a linear economy to a sustainable, circular bioeconomy based on bioresource recovery, green energy and methodologies with a zero-waste production [47,48]. Therefore, the application of FW and by-products as raw materials for protein recovery could lead us to wisely find sustainable solutions for their full exploitation in a circular economy approach.

## 5. Polyelectrolyte Precipitation vs. Traditional Extractive Methods

Proteins are present in plants, especially fruit and vegetables, as well as animal tissues; they are linked to structural and phyto-molecules such as polysaccharides, sugars and fibers. In this regard, it is essential to break down the intramolecular linkages and weaken and disintegrate cell wall structures to maximize the extraction and recovery of proteins from these matrices [49]. Most of the protein extraction processes involve different steps, including sample disruption and homogenization, solvent selection, protein extraction and stabilization.

Traditional extractive processes such as alkaline treatments (ATs), inorganic and organic solvents precipitation (IOSP) and liquid chromatography (LC) are the most widely used methods for protein recovery; although they are consolidated methods and have been extensively used during many years, they are currently categorized as nonsustainable with restricted industrial application [17,50]. For example, the ATs are limited by their low protein recovery yield (~30%), denaturation of protein, loss of important amino acids and time-consuming process, and the use of a high concentration of alkali solution represents a non-eco-friendly method. Although the IOSP methods employ a high water content which results in an advantageous situation for protein solubility and stability, the saturation levels (up to 60%) of the inorganic salts (ammonium sulphate) and high volumes of organic solvents (acetone, butanol, iso-propanol and ethanol) possibly lead to a toxic final product, and both precipitation processes are not protein selective, dealing with an extra step process for protein cleaning and purification to remove unwanted molecules from the protein solution [51]. The LC methods are well-recognized for assuring the recollection of target proteins with a high purity degree (≥90%), which allowed it to be directed to cosmetic or pharmaceutical industries; however, they still have some disadvantages to overcome, regarding a laboratory-intensive process, low production volume, time-consuming procedure and expensive equipment [52]. Having these unfavorable facts in mind regarding protein recovery techniques, the development of innovative and disruptive methods is manifestly needed. Therefore, ionic precipitation using Polys is a well-studied interaction methodology that, through these few years, have been used as an extractive technique and a sustainable green process, representing a nontoxic, low-cost and easy-to-scale process at the industrial scale to recover proteins in an efficient and feasible manner, conserving their structure and biological activity [9].

As described in Section 2, Polys can interact with solubilized proteins by the shift of pH or ionic strength, Poly–Pro ratio, polysaccharide linear charge density, protein surface charge density and stiffness of the polysaccharide chain, allowing the formation of soluble or nonsoluble complexes to take place and developing a semisolid hydrocolloid mixture between polyelectrolyte and proteins, which then is collected by centrifugation or decantation [10,53]. Such interactions are strongly conducted mainly by the functional groups (e.g., carboxylic and sulphonic) present in the structure of the Polys. In many of the cases, interactions between charged amino acids (cations) from proteins and anionic groups from Polys lead to typically natural Coulombic attraction forces as well as strong, but reversible, electrostatic and dipole–dipole interactions and hydrogen linkages [54]. Carrageenan (and derivatives), pectin, chitosan, alginate and Arabic gum are Polys naturally obtained from certain types of plants, fruits, red seaweed and vegetables, which are more preferred to be used than synthetic polyelectrolytes (polyacrylic acids and polystyrene) due to their nature of being nontoxic, biodegradable and water-soluble polysaccharides. These polymers are widely used in cosmetic, pharmaceutical and food industries due to their organic label categorization.

The main advantages to apply ionic precipitation is that the precipitated mixture (Poly–Pro complex) can be easily resolubilized in aqueous or buffer solutions (even in less volume if desirable) by pH or ionic strength modifications and soft mixture, as well as represent a nontoxic final product. Due to all aforementioned characteristics, the resulting product can be applied by different typologies of industries targeting human consumption [55].

Overall, to apply this protein recovery method, it is important to consider the next practical information (See Figure 2): (I) identify the specific characteristics of the target protein in terms of molecular mass, electric charge and isoelectric point; (II) select a polyelectrolyte with an opposite electric charge to the target protein; (III) as a first step, prior to advancing to proteins present in complex products, it is preferable to work first with protein standards, to verify and estimate the favorable interaction and precipitation conditions (pH interval and the minimum concentration of polyelectrolyte required to form the complex); and (IV) the target molecule must be assessed for its solubility, biological activity, structural form and the impact on thermal stability.

## 6. Innovation on Protein Recovery by Polyelectrolyte Precipitation—Case Studies

A diverse number of synthetic Polys have been employed for the recovery of several target proteins. However, natural Polys are preferable to be employed since they are sustainable precipitant agents and have shown similar and even better results than the synthetic ones. Data from recent studies have shown positive outcomes on the precipitation process with both synthetic and natural Polys mainly on their recovery yield and purification factors of proteins from diverse sources (Table 1). For example, polyacrylate, a negatively charged polyelectrolyte, and its derivatives (Eudragits L100 and S100) were used at a low concentration (0.002% *w*/*v*) and a slightly acidic pH (4.5) for the obtention of pancreatic serine proteases and peroxidases (positively charged proteins) from bovine pancreas and radish roots, respectively, resulting in an increase of 5-fold with a recovery of 33% of proteases and 1.5-fold with 50% of peroxidases [7,56]. Later, a natural Ɩ-carrageenan (negatively charged polysaccharide) at ≤0.005% (*w*/*v*) was employed to precipitate chymotrypsin at a pH of 4.5 [57]. An interesting comparative study between a negatively (poly vinyl sulfonate) and positively (chitosan) electric-charged polyelectrolytes was conducted by Boggione et al. [58] for the precipitation of a positively charged endoglucanase at pH 2.7 produced by microbial fermentation and its commercial form, obtaining better results by applying chitosan at a lower concentration (0.05% *w*/*v*) and higher pH (5.1) than poly vinyl sulfonate (1% *w*/*v*), with a purification factor of 7-fold and ~30% of recovered bioactivity. Another innovative research work was carried out by precipitating xylanase produced by fungal solid-state fermentation. Such a production system can generate very homogeneous extracts between target proteins, sugars and organic acids, among other interferences. Size exclusion and ion exchange chromatography (time-consuming and costly methodologies) are the most-used methods to recover and purify proteins. However, in this study, carrageenan and chitosan were effectively used to selectively recover xylanase with titles of purification factor of 9-fold (at 0.5% *w*/*v*, pH 7.00) and 5.6-fold (at 0.05% *w*/*v*, pH 8.00), respectively [59]. As it was previously stated, proteins are very important market products and useful industrial biomolecules; however, their production and obtention processes in some cases do not meet the consumer demands and concerns. Therefore, the obtention of new natural sources for the recovery of proteins is preferred. In this regard, Portugal and its transition to a circular economy by FW and by-product valorization is boosting the scientific and enterprise areas to look for plant-based proteins. For example, pineapple and its by-products (peels and cores) are already known for their enzymatic content, specifically in bromelain enzyme, with anti-inflammatory and proteolytic activities. Its purification process was already optimized by polyelectrolyte precipitation, remarking less carrageenan concentration usage (0.2–0.3% *w*/*v*, pH 5.1) with a high recovery yield of enzymatic activity (80 to 90%) [60]. Additionally, by the uniqueness and simplicity of this bromelain extraction process, the same researchers were awarded with an international patent in Europe (EP 3 252 156 A1) [61]. Following this subject matter, another research work was carried out for the extraction of a poorly explored enzyme from melon by-products (peels) called cucumisin with proteolytic and milk-clotting properties. The precipitation process was conducted by applying carrageenan at a lesser concentration (0.003–0.006% *w*/*v*, pH 3), obtaining better purification factors on proteolytic and milk-clotting activity (2.11- and 17.65-fold, respectively), when compared with the ammonium salt precipitation (1.60- and 2.06-fold, respectively) as well as a high recovery yield of enzymatic activity (60–80%) [62].

In summary, based on the good results and insights attributed by the application of polyelectrolyte precipitation and its favorable patent applications and developments as a measure of technological innovation, it could be suggested that this technique not only represents an eco-friendly extractive method but also a promising technology to generate new business lines and be transferred to pilot or industrial levels for the obtention of bioactive proteins with high market demands.

## 7. Future Perspectives on Proteins Recovery toward Circular Economy

From the last 5 years, a surprising increased awareness on proteins for developing novel, super and functional foods has arisen globally not only for researchers but also for business developments [65]. Plant-based proteins are more sustainable nutritional ingredients than animal-based proteins for the development of healthy foods to satisfy vegan/flexitarian/vegetarian and nutrition-conscious consumers, together with the increasing awareness of sustainable production and consumption [66]. However, as the plant-based protein industry is still at a high demand for innovation in technologies, processes and products, there is still the need to be a market segment that is effective regarding profitability and improvement of consumers’ literacy. Thus, to address the current market necessities, a considerable amount of investment must occur to improve the quality of ingredients’ functionalities and end-products as well as the application of novel green technologies, enabling a move forward to develop competitive and long-term feasible businesses.

There is a demand to exploit and develop optimized techniques important for novel plant-based protein recovery such as precipitation processes by Polys [10]. To highlight this subject matter, this extraction technique is found to be a promising method for isolation of enzymes from plant-based by-products, among other sources, conserving their native structural and functional conformation with a wide range of specific activities. Additionally, the integral application of this innovative technology for the recovery of proteins is gaining interest due to its simplicity and null use of sophisticated equipment [11]. Moreover, this method could provide higher yield, less time consuming, eco-friendly and lesser solvent consumption over the conventional methods. However, the deep characterization and optimization of this technique must be carried out when applied to different food waste proteins to obtain safe and reproducible final products. On the other hand, having in mind all the positive benefits coming from plant-based proteins, the search to look for more resources of proteins and their functionalities is a scientific task that must be addressed in parallel with enterprises in order to reach real and tangible results for society, sustainability and the economy [67].

## 8. Conclusions and Final Remarks

The consumption of non-animal-origin proteins is not only a trend but also a modern lifestyle, representing a vital long-term solution against many feed disorders and associated health issues that today’s society is facing. Although the research of this kind of proteins has already started and there is much evidence highlighting FW and by-products as novel alternatives and sources of these biomolecules, the real challenge is still the development and application of innovative and green technologies for their recovery. Therefore, protein precipitation by polyelectrolyte represents an integral emerging eco-innovative method focused on sustainable protein obtention without compromising the environmental sustainability. As discussed in this review article, the combination of the use of food waste or by-products as sources of proteins with the polyelectrolyte precipitation method for their extraction, symbolizes a promising integrative process to (i) valorize food waste, (ii) obtain a diversified pool of proteins/enzymes with high industrial interest and (iii) promote sustainable and circular economic growth. However, the good outcomes from many of the research works reported on this integrative process are often simply reported at the laboratory scale; for now, it is troubling to achieve investigations at an industrial scale, since it is necessary to optimize crucial parameters influencing their design and applications such as quantity, homogeneity, temperature and protein characteristics; such variables are vital for industrial consolidation and thus, need urgent attention. To conquer these lacks, the future direction of researchers and protein enterprises should converge and work in synergy to provide a deeper understanding of the relationship between protein structural changes and bioactive properties in relation to precipitation parameters at the laboratory and pilot levels to guarantee and maximize the application of these proteins in a real industrial scenario.

## Figures and Tables

**Table 1:** Spectrum of protein recovery from different sources by polyelectrolyte precipitation (natural vs synthetic).

Polyelectrolyte	Target Protein	Source	Conditions	Recovery Yield	Purification Factor	Reference *
Alginate and Carrageenan	Protein	Rice bran	Alginate-to-protein ratio of 1:1 and Carrageenan-to-protein of 2:1; pH 3.5	93% and 95% protein recovery, respectively	-	[63]
Carrageenan	Chymotrypsin	Bovine pancreas	0.06%, *w*/*v*; pH 4.5	60% enzymatic activity	3-folds	[55]
Carrageenan	Standard Chymotrypsin	Sigma Aldrich	0.005% *w*/*v*; pH 4.5	-	-	[57]
Eudragit L 100 and Eudragit S 100	Peroxidase	*Raphanus sativus* L.	0.002% *w*/*v*; pH 4	50% and 45% enzymatic activity	2-folds	[7]
Polyacrylate	Serine proteases	Bovine pancreas	0.05% *w*/*v*; pH 4.5	33% protein recovery	5-folds	[56]
Poly-vinyl sulfonate and Chitosan	Endoglucanase	Solid-state fermentation by *Aspergillus niger*	1% *w*/*w* and 0.05% *w*/*v*, respectively; pH 5.3	40% enzymatic activity	9- and 7-folds	[58]
Carrageenan	Standard Bromelain	Sigma Aldrich	0.08% *w*/*v*; pH 5.1	85–90% enzymatic activity	-	[17]
Carrageenan	Protein	*Chenopodium quinoa*	0.002–0.005% *w*/*v*; pH 2.9	-	-	[64]
Chitosan and Carrageenan	Xylanase	Solid-state fermentation by *Aspergillus niger*	0.05% *w*/*v*; pH 8.00 and 0.5% *w*/*v*; pH 7, respectively	40% and 30% enzymatic activity	6- and 9-folds	[59]
Sodium alginate	Lysozyme	Egg white	sodium alginate-to-protein ratio of 2:1 pH 3	97% protein recovery	-	[11]
Carrageenan	Bromelain	*Ananas comosus* Merr. peels and stem	0.2–0.3% *w*/*v*; pH 5.1	80–90% enzymatic activity	-	[60]
Carrageenan	Cucumisin	*Cucumis melo* L. peels	0.003% *w*/*v*; pH 3	90% proteolytic 60% milk-clotting activities.	2- and 18-folds	[62]

* References are cited in chronological order.
